# Identification of clear cell renal cell carcinoma and oncocytoma using a three-gene promoter methylation panel

**DOI:** 10.1186/s12967-017-1248-y

**Published:** 2017-06-29

**Authors:** Ana Sílvia Pires-Luís, Pedro Costa-Pinheiro, Maria João Ferreira, Luís Antunes, Francisco Lobo, Jorge Oliveira, Rui Henrique, Carmen Jerónimo

**Affiliations:** 1 Cancer Biology and Epigenetics Group, IPO Porto Research Center (CI-IPOP), Portuguese Oncology Institute of Porto (IPO Porto), Research Center-LAB 3, F Bdg., 1st Floor, Rua Dr António Bernardino de Almeida, 4200-072 Porto, Portugal; 2 Department of Pathology, Portuguese Oncology Institute of Porto (IPO Porto), Porto, Portugal; 3 Department of Epidemiology, Portuguese Oncology Institute of Porto (IPO Porto), Porto, Portugal; 4 Department of Urology, Portuguese Oncology Institute of Porto (IPO Porto), Porto, Portugal; 5 Department of Pathology and Molecular Immunology, Institute of Biomedical Sciences Abel Salazar (ICBAS), University of Porto, Porto, Portugal

**Keywords:** Kidney tumours, Renal cell tumour, Clear cell renal cell carcinoma, Oncocytoma, Epigenetics, Methylation, *OXR1*, *HOXA9*, *MST1R*, Diagnostic biomarker

## Abstract

**Background:**

Promoter methylation has emerged as a promising class of epigenetic biomarkers for diagnosis and prognosis of renal cell tumors (RCTs). Although differential gene promoter methylation patterns have been reported for the major subtypes (clear cell, papillary and chromophobe renal cell carcinoma, and oncocytoma), validation of diagnostic performance in independent series have been seldom performed. Herein, we aimed at assessing the diagnostic performance of genes previously shown to be hypermethylated in RCTs in different clinical settings.

**Methods:**

Promoter methylation levels of *HOXA9* and *OXR1* were assessed by quantitative methylation specific PCR. ROC curves were generated for *OXR1*, *OXR1* combined with *MST1R* and *HOXA9*. Sensitivity, specificity, positive predictive value, negative predictive value and accuracy were computed, maximizing specificity. Methylation levels were also correlated with clinical and pathological relevant parameters.

**Results:**

*HOXA9* and *OXR1* promoter methylation was disclosed in 73 and 87% of RCTs, respectively. A two-gene methylation panel comprising *OXR1* and *MST1R* identified malignancy with 98% sensitivity and 100% specificity, and clear cell renal cell carcinoma with 90% sensitivity and 98% specificity. *HOXA9* promoter methylation allowed for discrimination between oncocytoma and both papillary and chromophobe renal cell carcinoma but only with 77% sensitivity and 73% specificity. Significantly higher OXR1 promoter methylation levels (p = 0.005) were associated with high nuclear grade in ccRCC.

**Conclusions:**

A panel including *OXR1* and *MST1R* promoter methylation allows specific and sensitive identification of renal cell tumors, and, especially, of clear cell renal cell carcinoma. Moreover, higher *OXR1* promoter methylation levels associate with clear cell renal cell carcinoma nuclear grade, a surrogate for tumor aggressiveness. Thus, gene promoter methylation analysis might a useful ancillary tool in diagnostic management of renal masses.

## Background

Epigenetic deregulation is a frequent finding in renal cell tumors (RCT) [1]. These arise from renal cortical tubular cells and encompass several entities, the most frequent being clear cell renal cell carcinoma (ccRCC), papillary renal cell carcinoma (pRCC) and chromophobe renal cell carcinoma (chRCC), representing 75, 10–15 and 5% of all RCT respectively. These are malignant neoplasm, although of variable aggressiveness. Indeed, ccRCC and pRCC are those that most frequently progress through regional and systemic metastization, whereas chRCC is generally more indolent. Among benign tumors, the most frequent RCT is oncocytoma [2].

The differential diagnosis among specific RCT subtypes can be challenging, especially in those tumors composed of cells with granular eosinophilic cytoplasm, as some morphological and immunohistochemical overlap exists among oncocytoma, eosinophilic variant of chRCC and eosinophilic variant of ccRCC [2, 3]. However, since their prognosis is radically different, accurate discrimination among these entities is critical. Furthermore, RCT therapy is becoming progressively more conservative, especially those of small size, with increasing use of cryoablation or radiofrequency techniques [4], entailing the need for more accurate diagnosis in biopsy samples. Owing to the heterogeneity that characterizes RCTs, a small tumor tissue sample might impair an accurate diagnosis based on histopathological, histochemical and immunohistochemical features [4, 5]. In this context, epigenetic biomarkers may constitute a valuable ancillary tool for diagnosis in biopsies from renal masses.

Among epigenetic alterations, aberrant promoter methylation, which generally entails gene silencing [6], has emerged as a promising class of biomarkers in urologic neoplasms [7], including RCTs [8, 9]. Although several genes are known to be hypermethylated in RCTs, mostly in ccRCC [10], frequencies vary, with most genes displaying intermediate (20–70%) methylation frequencies. Among genes with consistently high (>70%) methylation frequency in RCC, *APAF1* [11, 12], *MDR1* [13], and *PTGS2* [13], should be highlighted (97–100, 86 and 94%, respectively). Recently, we showed that *MST1R* was also frequently methylated in RCC, and promoter methylation levels discriminated ccRCC from the remaining RCT subtypes with high specificity [14].

Nevertheless, over the last years, several high-throughput studies on RCC promoter methylation using an array-based approach, identified several other hypermethylated genes in RCC, which might be useful as diagnostic biomarkers. This somewhat extensive list includes *SPINT2* [15], *IGFBP1*, *IGFBP3*, *COL1A1* [16], *UCHL1* [17], *CXCL16*, *KTN19* [18], *IGFBP2*, *SOX17*, *COL1A2*, *BMP4*, *FRZB*, *TAL1*, *MCM2*, *KCNK4*, *HOXC6*, *CCNA1*, *HOXA11*, *TERT*, *TMEFF2*, *PGF*, *ZNF215*, *SMARCB1*, *TWIST1*, *IGFBP7* [19], *BNC1*, *COL14A1*, *SFRP1* [20], *PCDH8*, *CCDC8*, and *FBN2* [21]. Most of these studies, however, mostly focused on ccRCC, only, whereas other included the most frequent RCTs and identified specific methylation patterns for each subtype in general [22], or specifically for the distinction between chRCC and oncocytoma [23]. Still, diagnostic performance analysis of these putative RCT biomarkers has not been performed. Thus, we aimed to evaluate the diagnostic performance of promoter methylation of several genes previously identified as candidate RCT biomarkers in array studies [22, 23], including also *MST1R* [14], in several differential diagnosis scenarios.

## Methods

### Patients, sample collection and DNA extraction

Representative tumor tissue was collected from 120 patients, submitted to radical or partial nephrectomy at the Portuguese Oncology Institute of Porto (Portugal) between 2003 and 2007, comprising ccRCC, pRCC, chRCC and oncocytoma (30 cases of each). Additionally, morphologically normal kidney (cortical) tissue from 9 nephrectomy specimens for upper urinary tract neoplasia were also collected and served as controls.

Tissue samples were snap-frozen immediately after surgery, stored at −80 °C and subsequently cut in a cryostat. The presence of at least 70% of tumor cells in the sections was assessed in H&E stains. Genomic DNA extraction was performed as previously described [24]. Briefly, 10% SDS was added to the sample, then proteinase K (20 mg/mL, overnight, 55 °C) to digest DNA, followed by extraction with phenol–chloroform and precipitation with 100% ethanol.

Formalin-fixed paraffin-embedded routine sections were used for routine tumour classification and grading (WHO) as well as staging (TNM) [2]. Relevant clinical data was retrieved from clinical charts.

This study was approved by the Institutional Review Board (Comissão de Ética para a Saúde) of Portuguese Oncology Institute of Porto, Portugal (CES518/2010).

### Gene selection

*MST1R* (GenBank: NM_002447) promoter methylation was previously identified by our group through EpiTect Methyl II qPCR array (SABiosciences, Qiagen, Frederick, MD, USA), and proved to be a specific diagnostic biomarker for ccRCC [14]. Briefly, 20 samples (4 ccRCC; 4 pRCC; 6 chRCC; 6 oncocytoma) were tested with the EMT commercial assay (Cat. No. 524EAHS-901ZA-24) on a 7000 Sequence Detection System (Applied Biosystems, Foster City, CA, USA) according to manufacturer instructions. *MST1R*, the gene with the highest percent of hypermethylated DNA (representing the fraction of input DNA containing at least two methylated CpG sites in the targeted region) was selected for further analysis, and proved to be a specific ccRCC biomarker [14].

Relevant literature was also reviewed, focusing on methylation array studies comparing different RCT subtypes, and two additional genes were selected: *OXR1* (GenBank: NM_001198532.1), proposed as a promising diagnostic biomarker for proximal tubule-derived RCC (ccRCC and pRCC) [22]; and *HOXA9* (GenBank: NM_152739.3) owing to its potential to distinguish chRCC from RO [23].

### Bisulfite treatment

Bisulfite treatment to convert unmethylated cytosine to uracil, maintaining methylated cytosine as such, was performed with EZ DNA Methylation-Gold Kit (Zymo Research) according to the manufacturer’s instructions, in 169 fresh-frozen samples: 9 morphologically normal kidneys, 30 ccRCC, 30 pRCC, 30 chRCC, 30 RO, 20 bladder urothelial carcinoma and 20 prostate adenocarcinoma samples.

### Quantitative MSP—fresh-frozen tissues

Primers for the candidate genes were designed to amplify methylated bisulfite converted complementary sequences, using Methyl Primer Express v 1.0 (Applied Biosystems, Foster City, CA, USA), considering the best predicted primer pair for the promoter region of each gene. Primers are listed in Table 1. A reference gene (β-*actin*) was used to normalize for DNA input in each sample.

**Table 1:** Primer sequences used in quantitative methylation specific PCR for candidate genes

Primer set	Sense primer sequence (5′–3′)	Antisense primer sequence (5′–3′)
*MST1R* ^a^	AGCGTTAGTGTATAGCGGC	TAAACAACGATCCCGACA
*OXR1*	TTCGTTGTATATATCGAACGGT	CCGTACTAAATATCTCGTTAACT
*HOXA9*	TATTTAGTCGGTATTCGC	ACCTCGAACGCTTCCAT

^a^ *MST1R* promoter methylation primers from [14, 25]

For fresh-frozen tissues, quantitative real-time polymerase chain reaction (qMSP) was performed in an 7500 Real-time PCR system (Applied Biosystems, Foster City, CA, USA), after DNA bisulfite treatment, in a reaction volume of 20 µL consisting of 10 µL of SYBR ^®^ Green PCR Master Mix (Applied Biosystems, Foster City, CA, USA), 7 µL of H_2_O, 0.5 µL of forward primer, 0.5 µL of reverse primer and 2 μL of bisulfate-modified DNA. Each sample was run in triplicate. In each plate, “no template controls” were included as a control for contamination, and a calibration curve was constructed with serial dilutions (1:5) of bisulfite converted universally methylated DNA at all CpGs (CpGenome Universal Methylated DNA; Millipore, Billerica, MA), to quantify the amount of fully methylated alleles in each reaction. The amplification reaction was carried out at 95 °C for 2 min, then 45 cycles of 95 °C for 15 s and annealing temperature (60 °C for all genes) for 1 min, followed by melting curve analysis.

For each sample, the relative level of methylated promoter DNA was determined by the ratio between the mean quantity obtained by qMSP analysis for each gene and the mean quantity of the internal reference gene (*ACTB*), multiplied by 1000 for easy tabulation, according to the formula: methylation level = (target gene/reference gene) × 1000.

### Statistical analysis

The frequency of methylated samples was determined for each RCT type, considering the highest value determined in the normal kidney tissue as cutoff. Median and interquartile range of methylation levels were also computed. Kruskal–Wallis non-parametric ANOVA followed by Mann–Whitney U test (with Bonferroni’s correction) for pair-wise comparisons were used to identify significant differences in methylation levels among RCT subtypes and association with standard clinicopathological variables. Spearman’s test was performed to ascertain correlation between age and methylation levels.

Methylation levels of *OXR1* and *MST1R* were combined using a logistical regression model by computing a new variable with the predicted values.

To assess the performance of promoter methylation levels as diagnostic biomarkers, receiver operator characteristics (ROC) curves were constructed by plotting the true positive rate (sensitivity) against the false positivity rate (1-specificity), followed by computation of the area under the curve (AUC). Cutoff values based on ROC curve analysis, prioritizing specificity and then sensitivity, were selected for calculation of sensitivity, specificity, positive and negative predictive values, and accuracy.

Disease specific survival (time between diagnosis and death for renal cell carcinoma), disease free survival (time between treatment and the first metastasis or local recurrence) and overall survival (time between diagnosis and death irrespective of cause) curves were constructed using the Kaplan–Meier method, with log-rank test (univariable analysis) and Cox regression analysis, for standard clinicopathological variables (age, gender, histological subtype, pathological stage) and methylation level of candidate genes. For this purpose, candidate gene methylation levels were classified as low or high using the 75th percentile methylation value of each gene as cutoff, and age, stage and histological subtype were dichotomized as age <75 vs ≥75, stage I and II vs stage III and IV and pRCC vs ccRCC.

Statistical significance level was set at p < 0.05 (two-sided). Analysis was performed using IBM ^®^ SPSS ^®^ Statistics for Windows, version 22.0 (SPSS, Chicago, IL, USA). Graphs were built using GraphPad Prism 6.0 software for Windows (GraphPad Software Inc., La Jolla, CA, USA).

## Results

### Promoter methylation analysis by qMSP

Tumor samples were categorized as *HOXA9* or *OXR1* methylated using the respective highest methylation ratio value observed in normal/control samples as cutoff (14.11 for *HOXA9* and 1577.45 for *OXR1*). Using these criteria, 73 and 87% of tumor samples were considered hypermethylated at *HOXA9* and *OXR1* promoters, respectively. Considering each subtype, the highest *HOXA9* promoter methylation frequency was found in oncocytomas (93%), followed by ccRCC (70%), chRCC (67%) and pRCC (60%), whereas the highest *OXR1* promoter methylation frequency was found in pRCC (93%), followed by ccRCC and oncocytoma (87%), and then chRCC (80%).

**Fig. 1:**
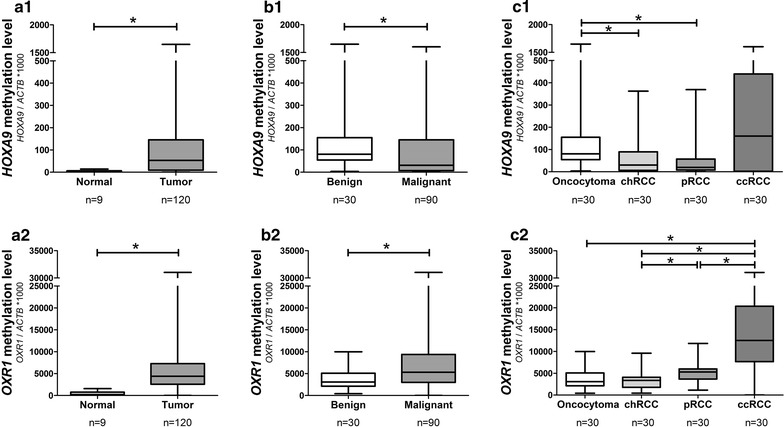
*OXR1* and *HOXA9* promoter methylation levels. Methylation levels in 9 normal renal tissue and 120 RCT samples for *HOXA9* (**a1**) and *OXR1* (**a2**); in 30 benign (oncocytoma) and 90 malignant (renal cell carcinoma) for *HOXA9* (**b1**) and *OXR1* (**b2**); and in the four RCT subtypes *HOXA9* (**c1**) and *OXR1* (**c2**). Target gene methylation level = target gene mean quantity/ACTB mean quantity * 1000). *chRCC* chromophobe renal cell carcinoma, *pRCC* papillary renal cell carcinoma, *ccRCC* clear cell renal cell carcinoma, *RCT* renal cell tumour

Levels in RCTs were significantly higher than in normal kidney (p < 0.001) (Fig. 1a). Moreover, *HOXA9* and *OXR1* promoter methylation also differed between benign (RO) and malignant (RCC) renal cell tumors (p = 0.011 and p = 0.009, respectively) (Fig. 1b). Among RCT subtypes, *OXR1* methylation were significantly higher in ccRCC compared to the remaining three subtypes (p < 0.001 for all), and also in pRCC compared to chRCC (p < 0.001) (Fig. 1c2). Concerning *HOXA9*, promoter methylation levels were only significantly higher in RO compared to chRCC (p = 0.004) (Fig. 1c1).

### Diagnostic performance of candidate biomarkers

**Fig. 2:**
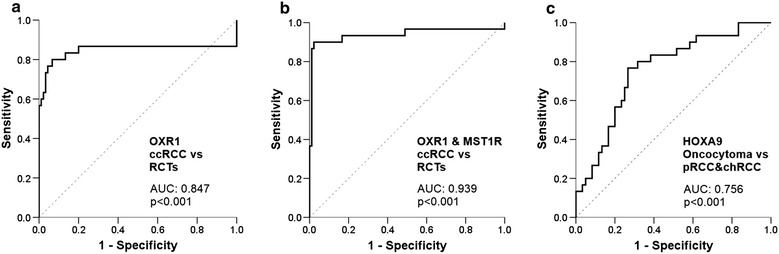
ROC curves for *OXR1, OXR1*&*MST1R* and *HOXA9* promoter methylation levels in different clinical settings. ROC curves for clear cell renal cell carcinoma (ccRCC) versus the remaining renal cell tumours (RCT) for *OXR1* (**a**) and for *OXR1*&*MST1R* (**b**), and for oncocytoma versus papillary (pRCC) and chromophobe (chRCC) renal cell carcinoma for *HOXA9* (**c**)

Both high *HOXA9* and *OXR1* promoter methylation levels discriminated normal from tumour samples with good sensitivity and high specificity (73 and 89% for *HOXA9* and 87 and 100% for *OXR1*, respectively) (Table 2). High *OXR1* promoter methylation levels also discriminated ccRCC from the remaining RCTs tested (pRCC, chRCC and RO) with 80% sensitivity and 93% specificity (AUC = 0.847) (Table 2; Fig. 2a).

**Table 2:** Diagnostic performance of *OXR1*, *OXR1*&*MST1R*, and *HOXA9* promoter methylation in different clinical settings

	SE (%)	SP (%)	PPV (%)	NPV (%)	Accuracy (%)
Normal vs tumour					
*OXR1* methylation	87	100	100	36	88
*OXR1*&*MST1R* methylation	98	100	100	75	98
*HOXA9* methylation	73	89	99	20	74
ccRCC vs RCT					
*OXR1* methylation	80	93	80	93	90
*OXR1*&*MST1R* methylation	90	98	93	97	96
RO vs pRCC&chRCC					
*HOXA9* methylation	20	95	67	70	70

*SE* sensitivity, *SP* specificity, *PPV* positive predictive value, *NPV* negative predictive value, *RCT* renal cell tumour, *ccRCC* clear cell renal cell carcinoma, *pRCC* papillary renal cell carcinoma, *chRCC* chromophobe renal cell carcinoma, *RO* renal oncocytoma

**Fig. 3:**
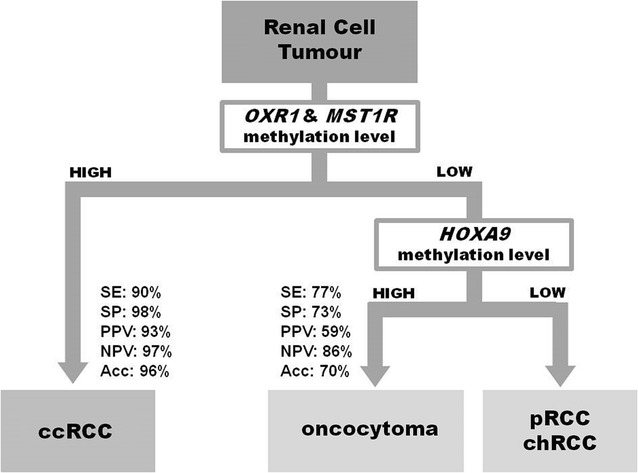
Proposed algorithm for discriminating among renal cell tumours in tissue samples. High promoter methylation levels of OXR1 combined with MST1R allowed identification of renal cell tumours (RCT) from normal renal tissue, and of clear cell renal cell carcinoma (ccRCC) from the remaining renal cell tumours (RCT). Then, a high promoter methylation level of HOXA9 allowed the identification of oncocytoma from papillary (pRCC) and chromophobe (chRCC) renal cell carcinoma. *SE* sensitivity, *SP* specificity, *PPV* positive predictive value, *NPV* negative predictive value, *Acc* accuracy

Considering these results and those that we previously reported for *MST1R* [14], a gene panel combining *OXR1* and *MST1R* gene promoter methylation was tested, and diagnostic performance increased for discrimination between ccRCC vs RCTs, displaying 90% sensitivity and 98% specificity (AUC = 0.939) (Table 2; Fig. 2b). Then, using *HOXA9* promoter methylation levels, RO could be discriminated from pRCC and chRCC with 77% sensitivity and 73% specificity (Table 2; Fig. 2c). A proposed combined use of these biomarkers is depicted in Fig. 3.

### Clinicopathological correlates

Relevant clinical and pathological data of the 120 RCT patients included in this study are depicted in Table 3 [14, 25]. The 9 patients from which normal kidney tissue was retrieved presented a median age of 69 years (range: 20–83), and 6 (67%) were males. No statistically significantly differences between RCT and normal kidney samples were found for age (p = 0.24) nor gender (0.453).

**Table 3:** Clinical and pathological features of the 120 RCT patients included in the study

	Tumour
Number of patients, n	120
Age, median (range)	60 (29–83)
Gender, n (%)	
Male	73 (61)
Female	47 (39)
Histological subtype, n (%)	
Clear cell RCC	30 (25)
Papillary RCC	30 (25)
Chromophobe RCC	30 (25)
Oncocytoma	30 (25)
Pathological stage, n (%)	
Stage I	47 (39)
Stage II	19 (16)
Stage III	21 (17.5)
Stage IV	3 (2.5)
n.a. (oncocytoma)	30 (25)
Nuclear grade, n (%)	
Grade 1	3 (2.5)
Grade 2	23 (19)
Grade 3	29 (24.5)
Grade 4	5 (4)
n.a. (chRCC and oncocytoma)	60 (50)
Metastasis during follow-up	
Clear cell RCC	9 (7.5)
Papillary RCC	7 (5.8)
Chromophobe RCC	1 (0.8)

*RCC* renal cell carcinoma, *chRCC* chromophobe renal cell carcinoma, *n.a.* not applicable

**Fig. 4:**
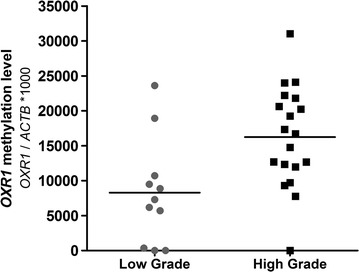
*OXR1* promoter methylation levels in clear cell renal cell carcinoma (ccRCC) according to nuclear grade. High grade (grade 3 and grade 4) clear cell renal cell carcinomas display higher *OXR1* promoter methylation level than low grade (grade 1 and grade 2) tumours (p = 0.005)

*OXR1* and *HOXA9* promoter methylation levels did not correlate with age (p = 0.08 and p = 0.18, respectively) or gender (p = 0.46 and p = 0.15, respectively). Considering all RCCs, methylation levels of *OXR1* and *HOXA9* were not associated with stage (p = 0.143 and p = 0.254 respectively) nor with the development of metastasis (p = 0.055 and p = 0.467 respectively). In ccRCC and pRCC, *OXR* (p = 0.008), but not *HOXA9*, promoter methylation levels associated with nuclear grade. Considering each histological subtype separately, higher *OXR1* promoter methylation levels were observed in high (3 and 4) grade tumours (median: 16,714; interquartile range: 11,993–21,817) compared to low (1 and 2) grade tumours (median: 7300; interquartile range: 355–10,715) in ccRCC only (p = 0.005) (Fig. 4).

No significant differences were found for age, gender or stage in ccRCCs that presented higher vs lower *OXR1* methylation levels (cutoff = median value of *OXR1* promoter methylation levels distribution, p = 0.486, p = 0.700 and p = 0.109, respectively).

### Survival analysis

During follow-up [median (range): 60 months (2–392 months)], 12 (13%) patients died from RCC and 17 (19%) developed metastatic disease.

Stages III and IV were associated with shorter cancer specific survival [HR: 13.5 (3–62), p = 0.001], disease-free survival [HR: 4.5 (1.7–12), p = 0.002] and overall survival [HR: 2.8 (1.3–6.1), p = 0.01] when compared to stages I and II, as expected. Considering only ccRCC and pRCC, the subtypes that most frequently display metastatic spread, pRCC was associated with shorter overall survival [HR: 2.7 (1.1–6.6), p = 0.033].

Higher *OXR1* or *HOXA9* promoter methylation levels were not associated with worse disease specific, disease free or overall survival.

## Discussion

Renal cell tumours, the most frequent (85–90%) kidney tumours, were classically diagnosed in advanced stage (IV), with large size, presence of metastasis and dismal prognosis when compared to kidney-confined tumours, which can usually be cured by complete surgical resection. However, with the increasing number of abdominal imaging studies performed due to unrelated symptoms, the number of incidentally diagnosed tumours has increased, posing new clinical challenges. These incidental tumours tend to be smaller (<5 cm) and kidney-confined, allowing complete surgical resection by partial nephrectomy in a high proportion of cases, or even alternative therapeutic strategies, including cryoablation or radiofrequency ablation. In these cases, renal mass biopsy is mandatory, for adequate risk stratification, which requires accurate diagnosis [4, 26]. However, despite tumour subtype identification being globally accurate (>90%) in renal mass biopsy, it is non-diagnostic in approximately 15% of patients, more frequently in small renal tumours. Moreover, it could underestimate tumour grade and stage in 25 and 5–10% of patients, respectively, and fail identification of pathologic features associated with aggressiveness (e.g., sarcomatoid differentiation) mostly due to sampling limitations [27]. In this context, diagnostic epigenetic biomarkers, including promoter methylation, might be clinically useful, mainly in patients considered for ablative techniques, in which renal mass biopsy is the sole available source of tumor material [27, 28]. Although several genes were consistently reported to be hypermethylated in RCC, and methylation array based studies reported different methylation patterns in distinct RCT subtypes [22, 23], validation in independent series has been seldom performed.

For this study, we selected three genes—*OXR1*, methylated in ccRCC and pRCC [22]; *HOXA9*, reported as differentially methylated in chRCC and oncocytomas [23]; and *MST1R*, highly methylated in ccRCC, that we previously shown to accurately identify ccRCC [14]—to assess their diagnostic performance in an independent series of 120 RCTs. Using robust methylation-specific primers for each gene promoter and performing quantitative methylation-specific PCR, we found that *OXR1* and *MST1R* promoter methylation discriminated between normal renal tissue and renal cell tumours with high specificity. Moreover, higher *OXR1* and *MST1R* methylation levels were characteristic of ccRCC (90% sensitivity and 98% specificity). Thus, this biomarker panel might be useful as ancillary diagnostic tool in renal mass biopsies with ambiguous morphological findings or limited tissue for microscopic evaluation. Furthermore, in patients with low *OXR1* and *MST1R* methylation level (NPV: 97%), *HOXA9* methylation level distinguished oncocytoma from chRCC and pRCC. This biomarker might be useful in cases in which Hale’s colloidal iron and immunohistochemistry (CK7, CD15) do not allow for a confident differential diagnosis between oncocytoma and chRCC [5]. This information might be clinically useful, not only to decide the best therapeutic strategy but also to select patients for active surveillance protocols [26, 27].

These results compare well with the reported performance of other DNA methylation-based biomarkers. *PCDH17* and *TCF21* promoter methylation identified renal cell tumours with 67% sensitivity and 100% specificity [29], but *OXR1* and *MST1R* were equally specific (100%) but more sensitive (98%) in the distinction between RCT and normal renal tissue. *PTGS2* was reported to distinguish ccRCC from the remaining RCTs subtypes with 46% sensitivity and 91% specificity [13], and we demonstrated that *OXR1* and *MST1R* reached a superior performance in all validity estimates. Moreover, *RASSF1A* hypermethylation was shown to discriminate pRCC from normal renal tissue with 87.5% sensitivity and 73.3% specificity, although comparison with other RCT subtypes was not undertaken [30]. Recently, an Illumina Infinium HumanMethylation450 (HM450) DNA methylation model for subtype prediction (encompassing angiomyolipoma, oncocytoma, ccRCC, pRCC and chRCC) that includes 59 variables (2 for angiomylolipoma, 9 for oncocytoma, 11 for normal kidney, 13 for ccRCC, 14 for pRCC and 10 for chRCC) was reported [31]. This model predicted for malignancy in 93% of samples, the correct subtype in 85% of RCT samples and 91% of ccRCC in the validation cohort (272 ex vivo core biopsies) [31]. Although we used a simpler and less expensive approach, correct ccRCC identification was reached in 98% of samples with the two-gene panel. Several studies focused on detection of aberrant promoter methylation in urine samples for RCT diagnosis. Nonetheless, the sensitivity was significantly lower than in tissue samples [29, 32, 33], and additional technical developments are warranted. Other epigenetic biomarker panels allowing for discrimination among RCT subtypes have been reported. A microRNA panel comprising miR-141 and miR-200b identified RCTs with high specificity and sensitivity (100 and 99%, respectively), discriminating oncocytoma from RCC and from chRCC with 86 and 90% sensitivity, respectively [34]. This performance is similar to that of *OXR1* and *MST1R* methylation panel for RCT (98% sensitivity, 100% specificity), although this panel did not perform as well concerning oncocytoma vs RCC in general.

Interestingly, some associations between promoter methylation levels and clinicopathological parameters were disclosed, although no impact in patient survival was apparent, probably due to the low number of events during follow-up. Indeed, high grade ccRCC displayed higher *OXR1* methylation levels than low grade ccRCC. This might be of clinical relevance as the most recently published biopsy series reveal high accuracy for RCT subtype identification, but poor reproducibility for tumour grading [4, 27]. Tumour grade is an important criterion for risk stratification of small renal masses, contributing for decisions about clinical management, i.e., either recruitment for active surveillance protocols, or selection for nephrectomy or ablative therapies [35]. Hence, a diagnostic biomarker that, in addition to histological subtype, also conveys information about tumor aggressiveness might improve risk stratification algorithms in biopsies from small renal masses.

The main limitations of this study concerns to the use of fresh-frozen tissue from renal tumours for molecular analysis, requiring validation in formalin-fixed paraffin embedded tissues from biopsy specimens before clinical implementation. Furthermore, the sensitivity and specificity for identification of oncocytoma requires improvement, eventually through the addition of another marker. Finally, the limited number of kidney cancer-related deaths and progression events impaired survival analysis.

## Conclusions

A panel including *OXR, MST1R* and *HOXA9* promoter methylation might be useful for positive identification of RCT, as well as for discrimination among subtypes. This panel could be used as ancillary diagnostic tool in the setting of renal mass biopsy, in which the amount of tissue available for histopathological examination may preclude a definitive diagnosis. Moreover, the panel might also improve risk stratification of patients harboring small renal masses, assisting clinicians in defining the best therapeutic strategy. Nevertheless, validation in larger independent cohorts is warranted to confirm the clinical potential of this gene methylation panel.

## Abbreviations

RCT: renal cell tumour
RCC: renal cell carcinoma
ccRCC: clear cell renal cell carcinoma
pRCC: papillary renal cell carcinoma
chRCC: chromophobe renal cell carcinoma
RO: renal oncocytoma
OXR1: oxidation resistance gene 1
HOXA9: homeobox A9
MST1R: macrophage stimulating 1 receptor
ROC: receiver operating characteristic
AUC: area under the curve
SE: sensitivity
SP: specificity
PPV: positive predictive value
NPV: negative predictive value
CNV: copy number variation
